# A key process of the nonstationary relationship between ENSO and the Western Pacific teleconnection pattern

**DOI:** 10.1038/s41598-018-27906-z

**Published:** 2018-06-22

**Authors:** Young-Hyang Park, Baek-Min Kim, Gyundo Pak, Masaru Yamamoto, Frédéric Vivier, Isabelle Durand

**Affiliations:** 1 Laboratoire LOCEAN-IPSL, Sorbonne Université (UPMC, Univ. Paris 6)-CNRS-IRD-MNHN, Paris, France; 2 Unit of Arctic Sea-Ice Prediction, KOPRI, Incheon, Korea; 3 Korea Institute of Ocean Science and Technology, Busan, Korea; 4 Research Institute for Applied Mechanics, Kyushu University, Kasuga, Japan

## Abstract

Recent studies have discovered an intriguing nonstationary relationship between El Ninõ–Southern Oscillation (ENSO) and the Western Pacific (WP) teleconnection pattern, one of the most prominent winter atmospheric circulation patterns in the North Pacific, with a regime-dependent interdecadal modulation of significant and insignificant correlations. However, the physical process underlying the observed nonstationary ENSO-WP relationship is a puzzle and remains to be elucidated, which is also essential for clarifying the still-debated nontrivial issue on whether the WP is directly forced by ENSO or by midlatitude storm tracks-driven intrinsic processes. Based on empirical orthogonal function (EOF) analysis of the upper-tropospheric teleconnection patterns and associated Rossby wave sources (RWS), we show that the nonstationarity in question is due to the regime-dependent constructive or destructive interference in meridional overturning circulation between the two leading EOFs of RWS best correlated with ENSO and WP, respectively. The observed insignificant correlation between ENSO and the WP after the 1988 regime shift can be explained by interrupted teleconnection between the tropics and high latitudes due to the collapse of the subtropical bridge pillar in the jet entrance region, consequence of the destructive interference. This suggested interference mechanism related to the regime-dependent upper-level RWS fields has significant implications for resolving the puzzle that hinders better understanding of decadal regime behaviors of the climate system in the North Pacific.

## Introduction

Atmospheric teleconnections, defined as significant simultaneous correlations in meteorological parameters at remote locations on Earth^1^, can be explained dynamically by the propagation of Rossby wave trains^2,3^, which act like an “atmospheric bridge” between different parts of the global climate system^4,5^. There are several prominent extratropical teleconnection patterns in the Northern Hemisphere, such as the North Atlantic Oscillation or its cousin Arctic Oscillation, and the Western Pacific (WP) and Pacific North American (PNA) patterns in the North Pacific^1,6^. Teleconnection patterns in the North Pacific in boreal winter (December-February) are primarily generated either by sea surface temperature (SST) forcing associated with El Ninõ–Southern Oscillation (ENSO) events in the equatorial Pacific Ocean or by internal dynamics associated with midlatitude synoptic eddies^1–7^. Other teleconnections such as the North Atlantic Oscillation^8^ and the Eurasian pattern^9^ could be partially forced by the North Atlantic SST, and the circumglobal teleconnection could be partially forced by the Indian Ocean SST^10^. A number of recent studies have emphasized a great implication of the WP pattern for the winter climate over East Asia and the northwestern Pacific^11–15^ as well as over central to eastern North America^16,17^.

However, the origin of these extratropical teleconnection patterns especially their connection to ENSO is not clear and has been the subject of debate in the literature. Based on a correlation analysis between the tropical time series and Northern Hemisphere geopotential height field together with the theoretical work on Rossby wave propagation on a sphere^2^, a pioneering study^18^ has attributed the origin of the WP and PNA to ENSO. In great contrast to this traditional view, studies since^19,20^ have claimed no direct connection of the WP to ENSO and attributed the former to eddy-driven intrinsic processes in midlatitudes^20^. An energetic perspective has also been proposed^21^ to support the internal dynamics of the WP pattern. Further complication in interpretation arises as the relationship between ENSO and the WP is not stationary but varies significantly with epochs. For example, consistent with the first report of the phenomena by Wang *et al*.^22^, a recent study^23^ has pointed out that during the period 1958–1976, ENSO exerted a strong impact on the North Pacific Oscillation (NPO), the surface expression of the WP^16^, while during the period 1977–2010 the ENSO-NPO relationship broke down. Another study^13^ has claimed that even during the latter period the relationship between ENSO and the WP is not stationary, with a significant correlation between them before the 1988 regime shift of the WP (correlation coefficient *r* = 0.68, null hypothesis probability *p* = 0.003 for 1973–1987), but practically no correlation after the regime shift (*r* = 0.14, *p* = 0.309 for 1988–2002). The exact physical process of such an intriguing nonstationary relationship is still unknown. However, the origin of decadal time scales frequently observed in relationships between pairs of different climate indices and parameters has been attributed to the interdecadal variability of the background states of the climate system^24,25^, although sampling variability^26^ could not be ruled out. There is also a speculation^13^ that the observed nonstationarity in the relationship between the WP and ENSO might be related to changing phases of Rossby waves due to the regime-dependent deepening or weakening of the East Asian trough near Korea. A more quantitative investigation of the ENSO-WP relationship is undertaken here by analyzing the wintertime upper-level circulation patterns and associated Rossby wave sources (RWS) generated by divergent flow^3^ (see Methods), demonstrating that the empirical orthogonal function (EOF) analysis of RWS is a powerful approach to separate the respective impact of ENSO and the WP on the meridional overturning circulation, and is thus crucial for quantitatively evaluating their regime-dependent interferences.

## Results

### Large-scale upper-level circulation patterns and associated storm track activity

**Figure 1:**
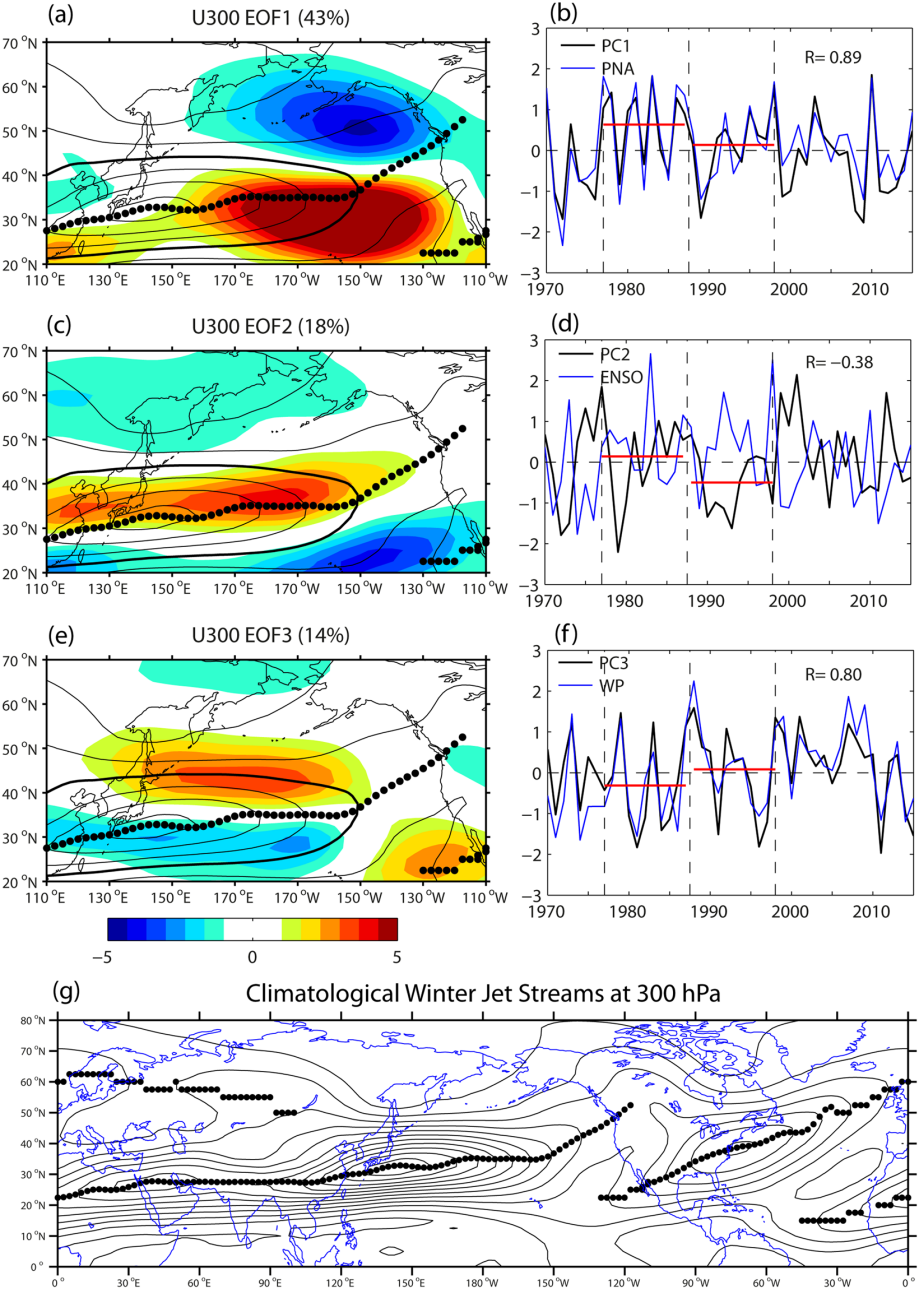
EOF analysis of winter-mean zonal wind at 300 hPa (U300) in the extratropical North Pacific sector. (**a**,**c**,**e**) Three leading EOFs of U300 (color, m s^−1^) superimposed on the climatological mean wind speed field at 10 m s^−1^ intervals (black lines), with the 30 m s^−1^ isotach shown thickened and maximum wind speed cores indicated by black dots. A hemispheric view of the latter field at 5 m s^−1^ intervals beginning from the 10 m s^−1^ isotach is given in (**g**). (**b**,**d**,**f**) The corresponding three PC time series in comparison with the best correlating climate indices: the PNA, ENSO, and WP, respectively. All anomaly time series are standardized with respect to their standard deviations. Red horizontal lines in (**b**,**d**,**f)** represent the time-mean PC values for two epochs before and after the 1988 regime shift of the WP. The superimposed maps are created by the MATLAB mapping package M_MAP v1.4 h using the m_coast function (https://www.eoas.ubc.ca/~rich/map.html).

Before presenting the EOF analysis of RWS in the North Pacific, it is instructive to briefly review the three leading EOF modes of the winter (December–February) 300 hPa zonal wind (U300) from the National Centers for Environmental Prediction–National Center for Atmospheric Research (NCEP/NCAR) reanalysis data^27^ for the 46-yr period 1970–2015 (see Methods). The first EOF (Fig. 1a), which explains a substantial fraction of the total variance (43%), exhibits a north-south dipole pattern straddling the climatological subtropical jet axis (black dots) in the central and eastern North Pacific. Its positive and negative poles are centered approximately at (30°N, 150 W) and (50°N, 150 W) in the jet exit region where the jet axis, albeit much weakened, bends sharply northeastwards. It is noteworthy that there is a discontinuity in jet axis along the west coast of North America where the northeastward bending jet axis disappears over the land near Vancouver and a new subtropical jet appears offshore Baja California (see Fig. 1g for a hemispheric view of jet axes). The leading principal component (PC) (Fig. 1b) corresponds closely to the PNA index (*r* = 0.89, *p* < 0.001), in agreement with previous works^28,29^. The second EOF (Fig. 1c) exhibits the strongest loading near the climatological jet core, with its PC2 (Fig. 1d) being moderately anti-correlated with ENSO (*r* = −0.38, *p* = 0.005). The third EOF (Fig. 1e) shows a north-south dipole pattern in the western and central basin, with a positive (negative) polarity at the northern (southern) flank of the climatological subtropical jet, which can be interpreted as a meridional shift of the jet. The corresponding PC3 (Fig. 1f) is highly correlated with the WP (*r* = 0.80, *p* < 0.001).

**Figure 2:**
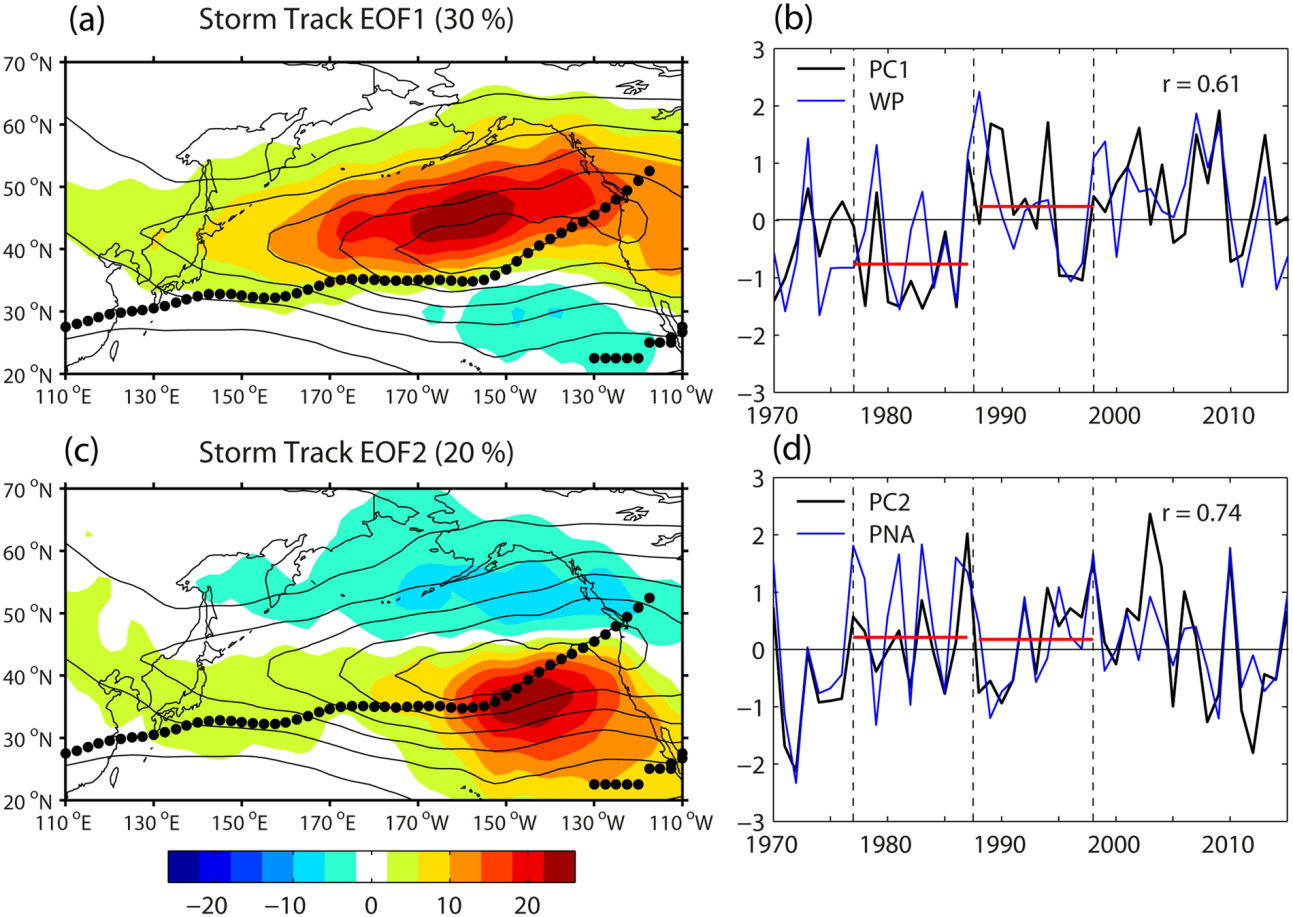
EOF analysis of storm track activity (STA) at 300 hPa in the extratropical North Pacific sector. (**a**,**c**) Two leading EOFs of STA (color, m^2^ s^−2^) superimposed on the climatological mean STA field at 20 m^2^ s^−2^ intervals (black lines), with the subtropical jet axis indicated by black dots. (**b**,**d**) The corresponding two PC time series in comparison with the best correlating climate indices: the WP and PNA, respectively. Red horizontal lines in (**b**,**d**) represent the time-mean PC values for two epochs before and after the 1988 regime shift of the WP. The superimposed maps are created by the MATLAB mapping package M_MAP v1.4 h using the m_coast function (https://www.eoas.ubc.ca/~rich/map.html).

**Figure 3:**
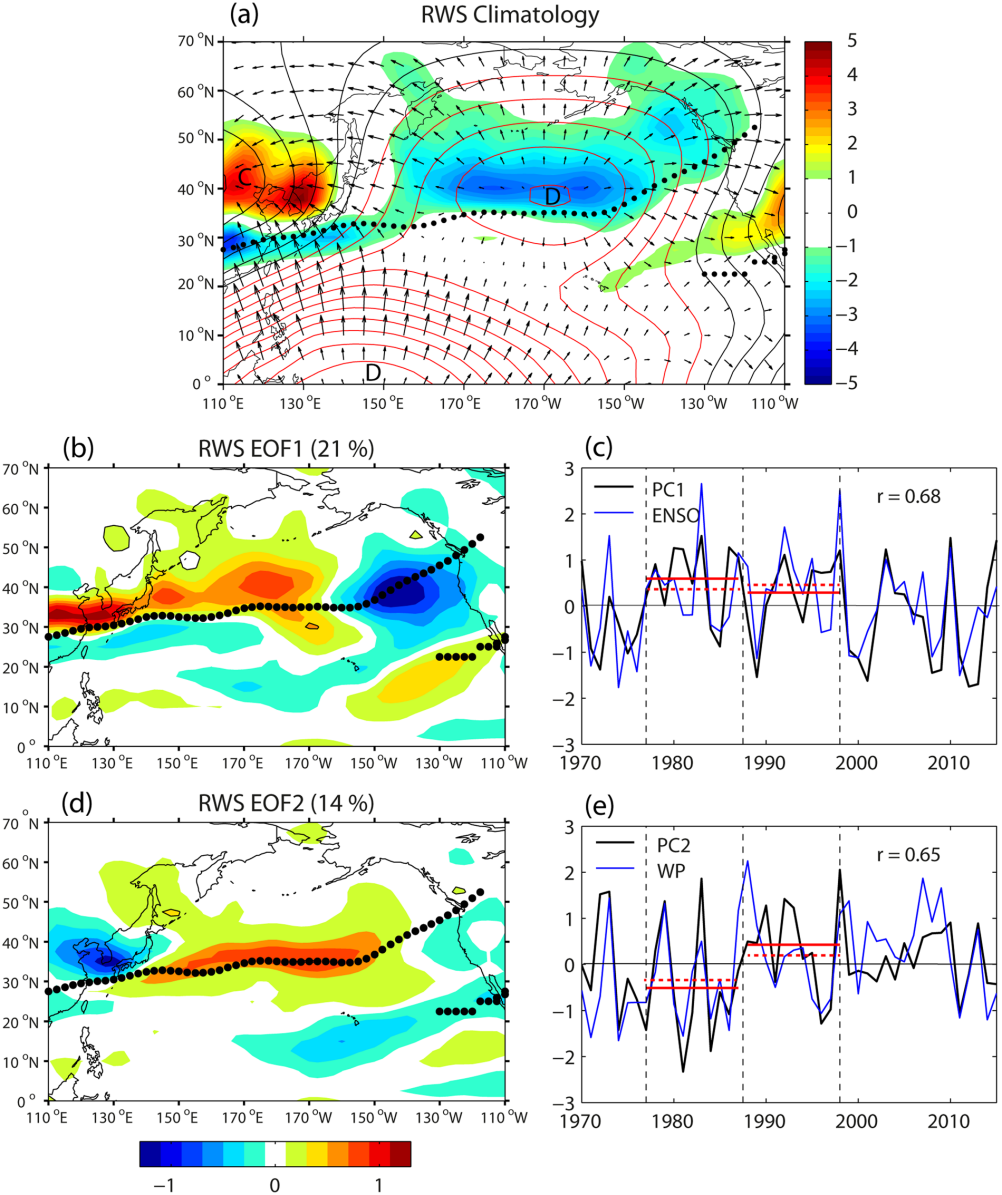
EOF analysis of Rossby wave source (RWS) at 200 hPa in the North Pacific sector. (**a**) Climatological RWS (color, 10^−10^ s^−2^) superimposed on the divergent wind field (vectors, arbitrary scale) and velocity potentials (red (black) lines for positive (negative) values, arbitrary scale). Centers of convergence and divergence are marked as C and D, respectively. (**b**,**d**) Two leading EOFs of RWS, with the climatological jet axis indicated by black dots. (**c**,**e**) The corresponding two PC time series in comparison with the best correlating climate indices: ENSO and WP, respectively. Red solid (dashed) lines in (**c**,**e)** represent the time-mean PC (ENSO/WP index) values for two epochs before and after the 1988 regime shift of the WP. The superimposed maps are created by the MATLAB mapping package M_MAP v1.4 h using the m_coast function (https://www.eoas.ubc.ca/~rich/map.html).

Regarding storm track activity (STA), which is estimated as the winter-mean variance of 2.5–6 day^4,7^ band-pass filtered daily meridional wind at 300 hPa, the two leading EOF modes are well correlated with the WP (*r* = 0.61, *p* < 0.001) and PNA (*r* = 0.74, *p* < 0.001), respectively (Fig. 2). EOF3 (figure not shown) is not significantly correlated with any teleconnection patterns discussed in this paper. EOF1 (Fig. 2a) projects mostly to the north of the subtropical jet axis along the northern flank of the climatological STA field whose core extends strongly eastwards along ~40°N. EOF2 (Fig. 2c) projects preferentially within the eastern basin, with a north-south dipole pattern straddling the northeastward-bending weakened jet axis. It is interesting to remark that the WP-related STA EOF1 (30%) dominates the PNA-related STA EOF2 (20%), which is in stark contrast with a minor contribution of the WP-related U300 EOF3 (14%, Fig. 1e) compared to the PNA-related U300 EOF1 (43%, Fig. 1a). Note also that the STA PC1 (Fig. 2b) exhibits an abrupt interdecadal jump across 1987/88, a period corresponding to the 1988 regime shift of the WP^13,30,31^. Such a regime shift is not observed with the STA PC2 neither with the PNA index (Fig. 2d). In addition, the STA PC1 is best correlated with the U300 PC3 (*r* = 0.51) and the STA PC2 with the U300 PC1 (*r* = 0.72). This information indicates that the STA in the North Pacific is primarily controlled by the latitudinal shift of the upper-level jet as seen in the U300 EOF3 (Fig. 1e) rather than by the jet strength (or baroclinicity) especially in the jet exit region as is evident in the U300 EOF1 (Fig. 1a). In fact, the intensified polar front jet along 43°N in the western Pacific as seen in the U300 EOF3 (Fig. 1e) roughly collocates with the subarctic oceanic frontal zone which acts to maintain the effective restoring of the atmospheric baroclinity^32^. This association of the anomalous polar front jet and the subarctic oceanic frontal zone leads to the efficient energy conversion for eddy growth, thereby providing the most favorable condition for generating enhanced STA anchored on the oceanic frontal zone^32^. Note however that the regime shift around 1988 is less evident in the U300 PC3 (Fig. 1f) as compared to that in the STA PC1 (Fig. 2b), as well as in the RWS PC2 as will be shown later (see Fig. 3e). This is probably related to the fact that the EOF3 of U300 is not statistically well separated from its EOF2 according to the North criterion^33^, although clearer evidence of the regime shift appears when rotated EOF analysis is performed (figure not shown). Finally, it is worth emphasizing that the WP can be considered to be largely eddy-driven considering its tight connection with the leading EOF mode of STA explaining as much as 30% of the total variance. However, there is also possibility that eddies are modulated by the WP in view of the triple association among the polar front jet, the subarctic oceanic frontal zone, and enhanced STA^32^. In fact, the STA and WP are known to vary simultaneously and mutually interacting, in tight association with one the other. Such a tight association has been previously coined as symbiotic relation^34^ between planetary and synoptic-scale waves in an equilibrated state of the atmosphere. The tight association of the STA and WP in a statistical sense has also been stressed by Trenberth *et al*.^4^ by noting that changes in quasi-stationary waves (such as the WP) engender changes in storm tracks, which in turn feedback to maintain or strengthen the quasi-stationary waves through vorticity and momentum transports.

### Upper-level Rossby wave sources and their association with teleconnection patterns

The wintertime RWS at 200 hPa is calculated with the NCEP/NCAR reanalysis data of divergent wind component according to the barotropic vorticity balance formulation^3^ (see Methods). The climatological RWS (Fig. 3a) is positive around Korea (~40°N, ~125°E) and to a lesser extent northeast of Hawaii; it is negative north of the jet axis in the central Pacific (35°–50°N, 150°E–130°W) and along the jet in the jet entrance region (30°N, 110°E–150°E), in agreement with a previous work^35^. The spatial distribution of climatological RWS is closely associated with the climatological divergent wind field, which shows two major sources of upper-level divergence: one in the western tropical Pacific (though not associated with any significant RWS due to the proximity to the Equator) and the other in the midlatitude central Pacific having a large band of negative RWS. Divergent winds from these sources converge into the East Asian trough at 40°N near Korea, creating a distinctive positive pole, apart from a secondary convergence zone northeast of Hawaii at 20°–40°N. Also, the negative RWS extrema of the central Pacific coincide with the climatological STA maxima (Fig. 2a), implying that the RWS there is linked to the STA via upper-level divergent flow.

Two leading EOFs of anomalous RWS (Fig. 3b–e) show their prominent loading concentrating mostly in the vicinity of the climatological jet axis where absolute vorticity and its gradient are large. Spatial polarity changes of these leading EOF modes are remarkable especially over the East-Asian marginal seas region west of 150°E, similarly to the climatological RWS. EOF1 is well correlated with ENSO (*r* = 0.68, *p* < 0.001) and exhibits a zonal strip of positive polarity just north of the jet axis between 110°E and 160°W, which is paired with a minor negative band just south of the jet axis especially in the jet entrance region (Fig. 3b,c). In the eastern basin a well-developed negative pole is found in the jet exit region centered at approximately (40°N, 140°W). EOF2 is well correlated with the WP (*r* = 0.65, *p* < 0.001) and reveals a distinctive negative pole in the East Asian trough region centered at Korea (Fig. 3d,e). This is in great contrast with the zonally-elongated positive polarity of EOF1, extending from the East Asian coast to 160°W, as already mentioned. Also remarkable for the PC2 best correlated with the WP is clear evidence for the 1988 regime shift as detected from the Rodionov sequential algorithm method^36^, whereas the PC1 best correlated with ENSO does not show any significant interdecadal variability in the 1977–1998 period (see red solid lines in Fig. 3c,e), consistent with previous works^13,30^. As expected from their mutually significant link with the WP (Figs 3e,2b,1f), the RWS EOF2 is also significantly correlated with the STA EOF1 (*r* = 0.38, *p* = 0.005) and the U300 EOF3 (*r* = 0.74, *p* < 0.001).

**Figure 4:**
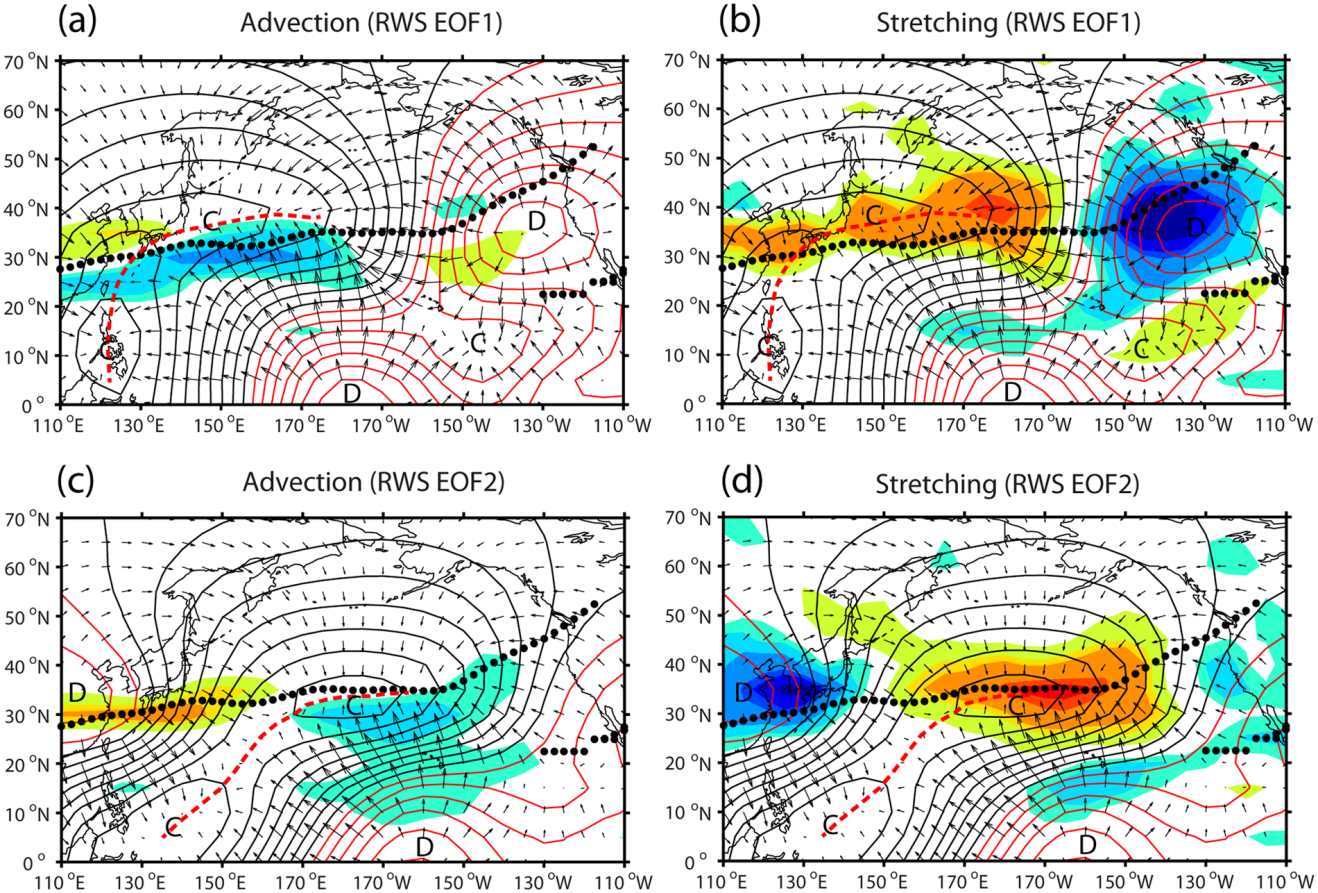
Decomposition of Rossby wave source (RWS). (**a**,**c**) Total advection term regressed onto the PC1 and PC2 of RWS, respectively. (**b**,**d**) Same as (**a**,**c**) but for the total stretching term. The anomalous RWS pattern for each term (same color scale as in Fig. 3) is superimposed on the divergent wind field (vectors, arbitrary scale) and velocity potentials (red (black) lines for positive (negative) values, arbitrary scale), both regressed onto the corresponding EOF mode of RWS. Centers of convergence and divergence are marked as C and D, respectively, with the climatological jet axis indicated by black dots. Red dashed lines represent extended regions of convergence. The superimposed maps are created by the MATLAB mapping package M_MAP v1.4 h using the m_coast function (https://www.eoas.ubc.ca/~rich/map.html).

To better understand its potential causes, the anomalous RWS is decomposed into the total advective and total stretching terms (see Methods), although the total stretching term is predominant for both EOF modes of RWS (Fig. 4). Also, the total stretching term is mostly determined by the anomalous divergence term (figure not shown), so we mainly refer to Fig. 4b,d to depict principal causes of the anomalous RWS. For EOF1 (Fig. 4b), there are two major centers of upper-level divergence; an equatorial center around the dateline without any significant RWS, which is likely related to deep convection caused by ENSO-related SST forcing. There is an extratropical divergence center showing a strong negative RWS in the eastern Pacific jet exit region (35°N, 140°W). This upper-level divergence is likely caused by transient eddy forcing^37^ associated with the STA because there is no midlatitude heating source generating deep convection comparable to that of ENSO. It is well known that midlatitude storms redistribute heat and momentum and thereby help to maintain low-frequency atmospheric circulation patterns^7,28^. The eddy heat and vorticity fluxes also induce rising motion poleward of the storm track and sinking motion farther equatorward^37^. In view of the association of rising (sinking) motion with upper-level divergence (convergence)^37,38^, a tropical convergence center over Philippines (10°N, 120°E) is likely associated with the subsidence branch of the Walker cell^38^. The midlatitude convergence region just north of the subtropical jet axis is characterized by an elongated zonal band of positive RWS, which likely manifests not only the subsidence branch of the thermally-driven local Hadley cell from the tropics but also those of the eddy-driven midlatitude zonal and meridional (local Ferrel-like) cells^38^ associated with the STA.

For EOF2 (Fig. 4d), the tropical Walker cell shifts eastward by 20°, with an equatorial divergence center at 160°W and a tropical convergence center at (10°N, 140°E) in the southern Philippines Sea. At 35°N, a divergence center is found in the East Asian trough region around Korea and a convergence center around the dateline, the latter being also well connected with an equatorial divergence centered at 160°W. Consequently, as inferred from red dashed lines in Fig. 4b,d, the extended convergence region associated with the tropical Walker and subtropical Hadley cells for EOF2 is shifted eastward by 20–30° compared to that for EOF1. Moreover, there appears in the East Asian trough region a striking phase contrast of RWS between EOF1 (positive/cyclonic) and EOF2 (negative/anticyclonic).

Although EOF1 and EOF2 of RWS are best correlated with ENSO and the WP (Fig. 3), respectively, they are also significantly correlated with the PNA (*r* = 0.54 for EOF1) and ENSO (*r* = 0.55 for EOF2). On the other hand, the WP and PNA teleconnection patterns result largely from midlatitude internal processes associated with the two leading modes of STA (Fig. 2). Taken together, above information implies that the two leading modes of RWS may represent the combined effects of atmospheric external forcing due to tropical ENSO events and midlatitude internal processes associated with the STA. Therefore, the combination of the two EOFs should represent the combined effects of ENSO and the midlatitude STA which is tied to the WP (PNA) pattern that prevails in the western (eastern) Pacific basin^1,6^. The two EOFs having opposite polarities in the East Asian trough region tend to reinforce (cancel) their respective contributions for 1977–1987 (1988–1998) when the time-mean PCs have opposite (same) signs (Fig. 3c,e). This is because the time-mean RWS field for a given EOF mode may be reconstructed by the EOF spatial pattern multiplied by the corresponding time-mean PC, such that only the time-mean field for EOF2 for 1977–1987 is reversed for its polarities from those seen in Fig. 3d.

### Tropical-extratropical teleconnection in terms of meridional overturning circulations associated with Rossby wave sources

**Figure 5:**
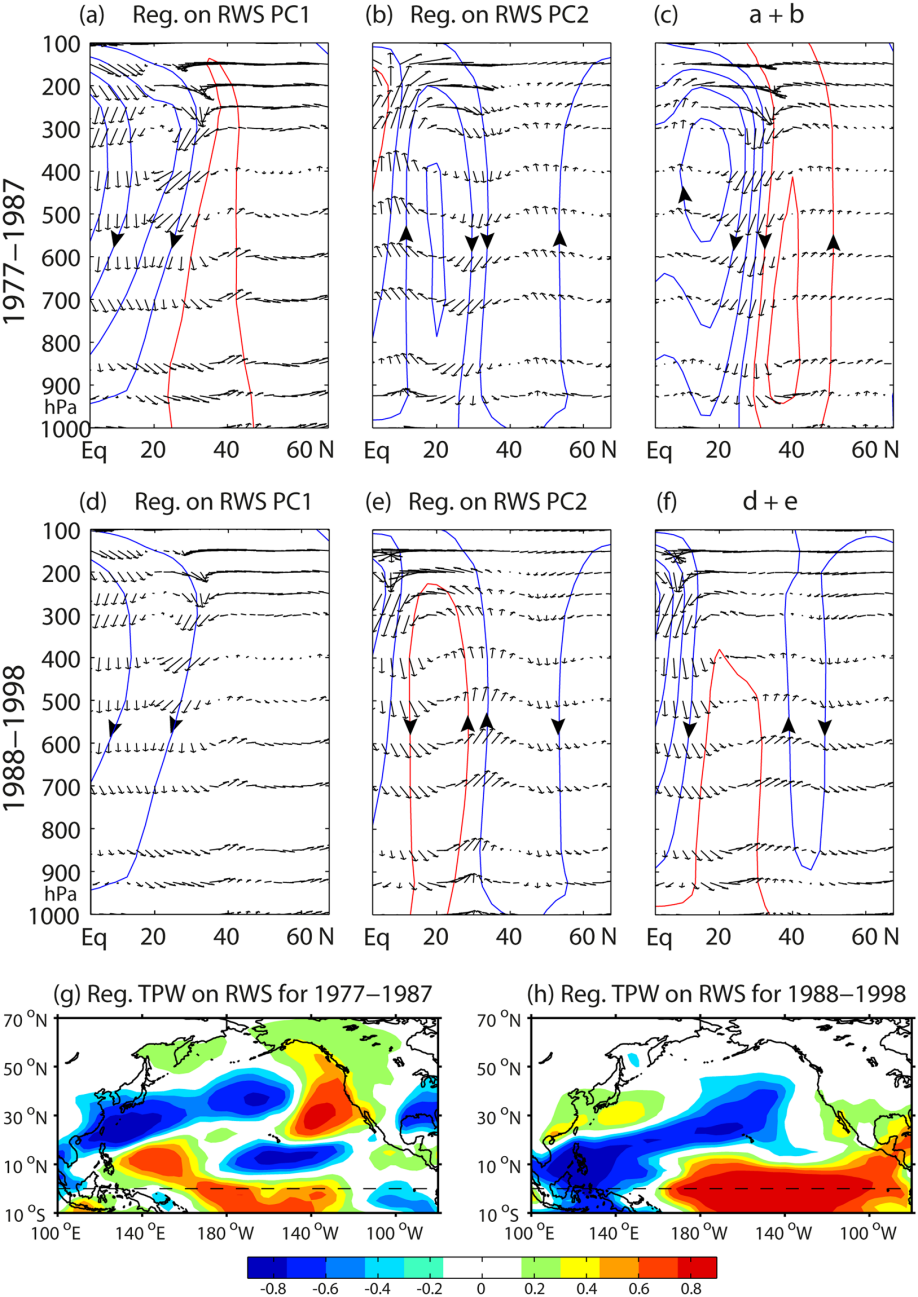
Meridional overturning circulations averaged over the western Pacific region (120°−160°E) and regressed onto the two leading EOF modes of RWS. Regression for the 1977–1987 epoch onto (**a**) the RWS PC1, (**b**) the RWS PC2, and (**c**) the sum of these two. (**d**–**f**) Same as (**a**–**c**) but for the 1988–1998 epoch. The arrows represent the divergent wind vectors in the meridional-vertical plane and the contours with large arrows are corresponding stream functions: red (blue) for anticlockwise (clockwise). Units are arbitrary. (**g**) Anomalous total precipitable water (TPW) (color, kg m^−2^) for 1977–1987, as obtained by the sum of the regressions onto the two PCs of RWS. (**h**) Same as (g) but for 1988–1998. The superimposed maps are created by the MATLAB mapping package M_MAP v1.4 h using the m_coast function (https://www.eoas.ubc.ca/~rich/map.html).

Distinguished horizontal pattern of upper-level RWS implies that there exists a corresponding local meridional overturning circulation, which can be used for scrutinizing the physical connection between the tropics and extratropics^38^, although there is a caveat that local overturning circulations are not closed circulations. In this sense the leading principal component of RWS is a useful index for measuring the relative intensity and direction of the overturning circulation. As we are interested in the ENSO-WP relationship, anomalous meridional overturning circulations in the western Pacific are obtained by averaging anomalous divergent winds and vertical velocities between 120°E and 160°E. The results are not sensitive to the choice of the averaging longitudes between 110°E and 170°E. Figure 5 shows the overturning circulations regressed onto the epoch-mean PC1 and PC2 of RWS as well as their sum, separately for two periods before and after the 1988 regime shift of the WP. See Methods for the epoch-mean regression.

**Figure 6:**
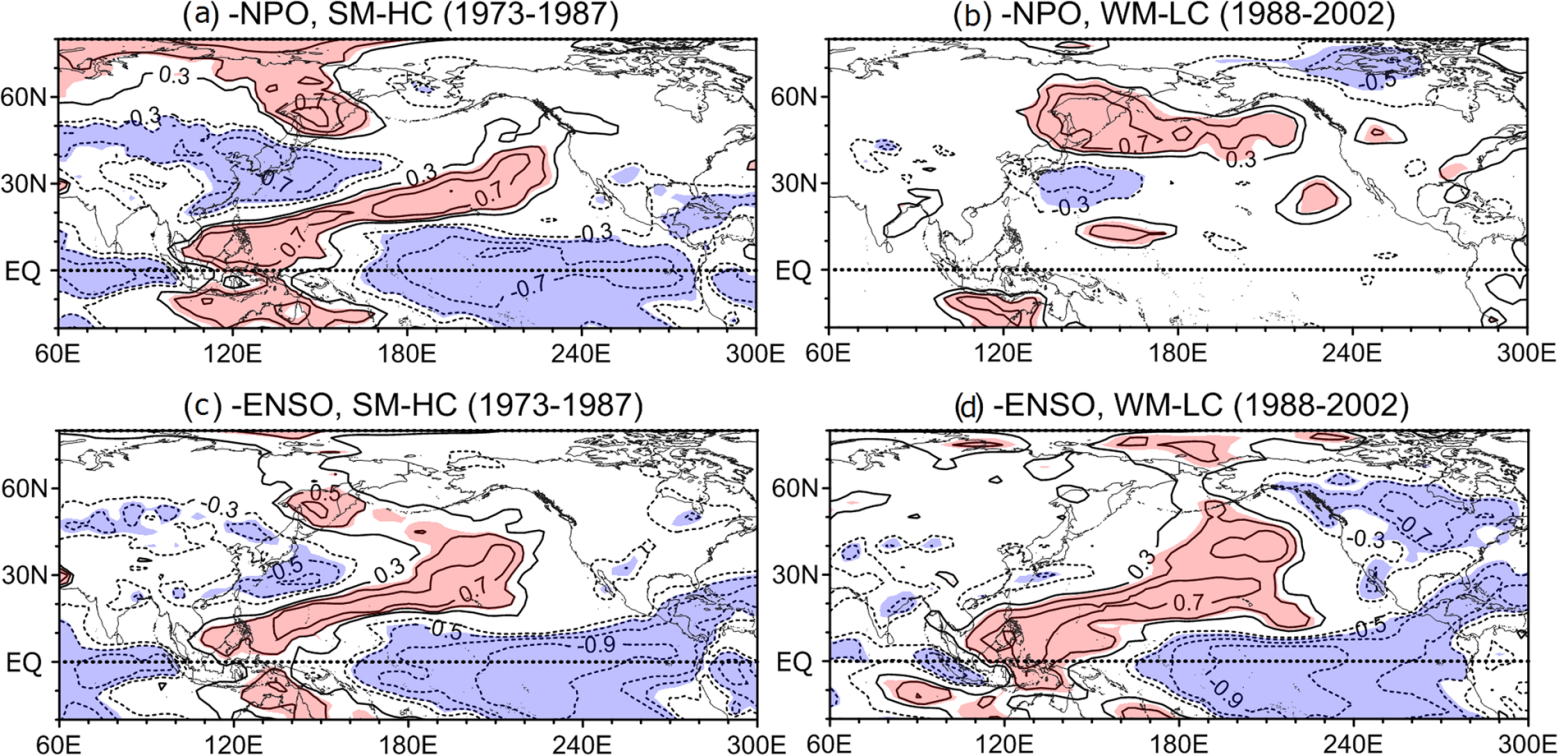
Maps of correlation between total precipitable water (TPW) and NPO/ENSO indices. (**a**,**c**) TPW vs –NPO and TPW vs. –ENSO for 1973–1987. (**b**,**d**) Same as (**a**,**c**) but for 1988–2002. Significant correlations at the 95% confidence level are stippled in red (blue) for positive (negative) values. Adapted from Fig. 14^13^. Used with permission of American Meteorological Society.

During the 1977–1987 pre-regime shift epoch, the epoch-mean regression of anomalous overturning circulation onto the RWS PC1 (Fig. 5a) reveals prominent subsidence in the tropical and subtropical regions south of 35°N, while the vertical motion is featureless to the north, especially in the upper-levels above 400 hPa. In contrast, the regression onto the RWS PC2 (Fig. 5b) reveals wave-like alternating clockwise and anti-clockwise overturning circulations, with tropical upward motion accompanied with upper-level divergence, subtropical subsidence accompanied with upper-level convergence, and subpolar upward motion accompanied with upper-level divergence. Due to the constructive (destructive) interference of subtropical (tropical) vertical motions, the sum of the two regressed circulations (Fig. 5c) reinforces the subtropical subsidence, while the tropical vertical motion much weakens. The strengthened subtropical subsidence is accompanied with enhanced upper-level convergence at 30°−35°N, which may serve as an effective “bridge pillar” creating a favorable condition for tightly connecting the subtropical Hadley-like and midlatitude Ferrel-like circulation cells. We mean by “bridge pillar” a junction connecting a thermally-driven Hadley cell^38^ and an eddy-driven Ferrel-like cell^37^ from a viewpoint of the atmospheric bridge. Referring to a teleconnection schematic of poleward-propagating upper-level Rossby wave trains^4^, the bridge pillar in the western Pacific corresponds to a cyclone (convergence/subsidence) in the East Asian trough region around Korea, via which a tropical anticyclone (divergence/ascent) in the Philippines Sea region can be teleconnected with a subpolar anticyclone (divergence/ascent) in the Kamchatka region. We recall that the Philippines Sea (Kamchatka) region locates close to the La Nina (northern pole) center of action for ENSO (WP^16^). Supporting evidence for this is given in Fig. 5g which shows to the west of 160°E three alternating-sign tropical, subtropical, and subpolar poles of total precipitable water (TPW), a measure of ascent (positive TPW) or subsidence (negative TPW)^13^. The anomalous TPW for 1977–1987 (Fig. 5g), obtained as combined regressions onto the two EOFs of RWS, shows a well-established tropical-extratropical teleconnection pattern in the western Pacific, with a tropical ascent in the Philippines Sea, a subtropical subsidence south of Korea and Japan, and a subpolar ascent at Kamchatka. This is also consistent with a previous work^13^ showing significant correlations between TPW and both the NPO and ENSO in the vicinity of the above three poles of teleconnection for an extended period of 1973–1987 (Fig. 6a,c). It is reminded that the NPO is the sea level footprint of the WP^16^. The well-established tropical-extratropical teleconnection pattern in the western Pacific, which results from the constructive interference of the two leading modes of RWS-related overturning circulations, can be interpreted as a plausible mechanism responsible for the observed significant correlation between ENSO and the WP before the 1988 regime shift.

In complete opposition to its overall negative state in 1977–1987, the RWS PC2 in the 1988–1998 post-regime shift epoch switches to an overall positive state (Fig. 3e), which leads to ascending motion in the subtropics and subsidence at both tropical and subpolar latitudes (Fig. 5e). In contrast, the regression onto the RWS PC1 (Fig. 5d) produces a much weaker stream function but with the same polarity of overturning circulation before and after the 1988 regime shift (Fig. 3c). Consequently, there is a large cancellation or destructive interference in the subtropics between the two regressed vertical circulations, whereas reinforced subsidence appears in the tropics (Fig. 5f). This break down of the subtropical bridge pillar makes the tropical and midlatitude overturning circulations disconnected. This is consistent with the anomalous TPW field for 1988–1998 (Fig. 5h) which shows in the western Pacific an opposite overturning circulation pattern (or a much weakened local Hadley cell) as compared to that for 1977–1987 (Fig. 5g), with a subtropical ascent (positive TPW) south of Japan, a tropical subsidence (negative TPW) over Philippines, and very weak subsidence poleward of 40°N. In contrast, a strengthened local Hadley cell occurs farther eastward in the central Pacific, with enhanced tropical ascent to the east of the dateline and associated strong subtropical subsidence near Hawaii. Therefore, in 1988–1998 the teleconnection from the tropics to the extratropics is suppressed in the western Pacific, while it is enhanced in the central Pacific. This is also nicely supported by the lack of significant correlations between the NPO and TPW in the western tropical Pacific around Philippines for an extended period of 1988–2002 (Fig. 6b), which is paired with a complete disappearance of significant correlations between ENSO and TPW in the northwestern Pacific poleward of 30°N (Fig. 6d). This interrupted teleconnection between the tropics and high latitudes due to the collapse of the subtropical bridge pillar around Korea may explain the observed insignificant correlation between ENSO and the WP after the 1988 regime shift.

## Discussions

EOF analysis of the upper-tropospheric RWS is shown to be a powerful means to separate the respective impact of ENSO and the WP, which are well projected onto the two leading EOF modes of RWS. These two EOFs reveal an opposite polarity between each other in the East Asian trough region around Korea and display quite different low-frequency variability, with an abrupt regime shift around 1988 for the EOF2 best correlated with the WP but without any significant interdecadal variability in 1977–1998 for the EOF1 best correlated with ENSO. The combination of such a different low-frequency variability of the two climate indices and the opposite polarity of their best correlated RWS in the East Asian trough region results in a constructive (destructive) interference between corresponding overturning circulations before (after) the 1988 regime shift. These regime-dependent contrasting interferences in overturning circulation, closely related to the different low-frequency variability of the background states of the two climate indices, appear as a key process responsible for the observed nonstationary ENSO-WP relationship. This interference mechanism via RWS suggests a first clue for unveiling the puzzle that hinders better understanding of decadal regime behaviors of the climate system in the North Pacific in relation to the tropical ENSO variability.

More specifically, the midlatitude eddy-driven overturning circulations and associated RWS are tied to the STA, which is in turn modulated primarily by the interdecadal variability of the WP, both showing the well-defined regime shift around 1988 as they are in symbiotic relation^4,34^. It is argued that the interdecadal changes in STA may induce the concurrent changes in RWS via eddy-driven secondary circulation^37^ especially in the East Asian trough region around Korea. As both the ENSO index and the RWS EOF1 do not reveal any polarity change around 1988 (Fig. 3c,e), the nonstationarity in the ENSO-WP relationship should be related to the regime shift of the RWS EOF2 best correlated with the WP, although the exact cause of the regime shift itself remains to be elucidated. To substantiate our findings obtained from the reanalysis data, it may be instructive to carry out in the future controlled model experiments to access the leading modes of RWS corresponding to different states of the base flow mimicking the interdecadal changes of the WP and ENSO. To our knowledge, the ENSO-WP relationship relevant to our subject has not been studied to any extent with a coupled ocean-atmosphere general circulation model.

**Figure 7:**
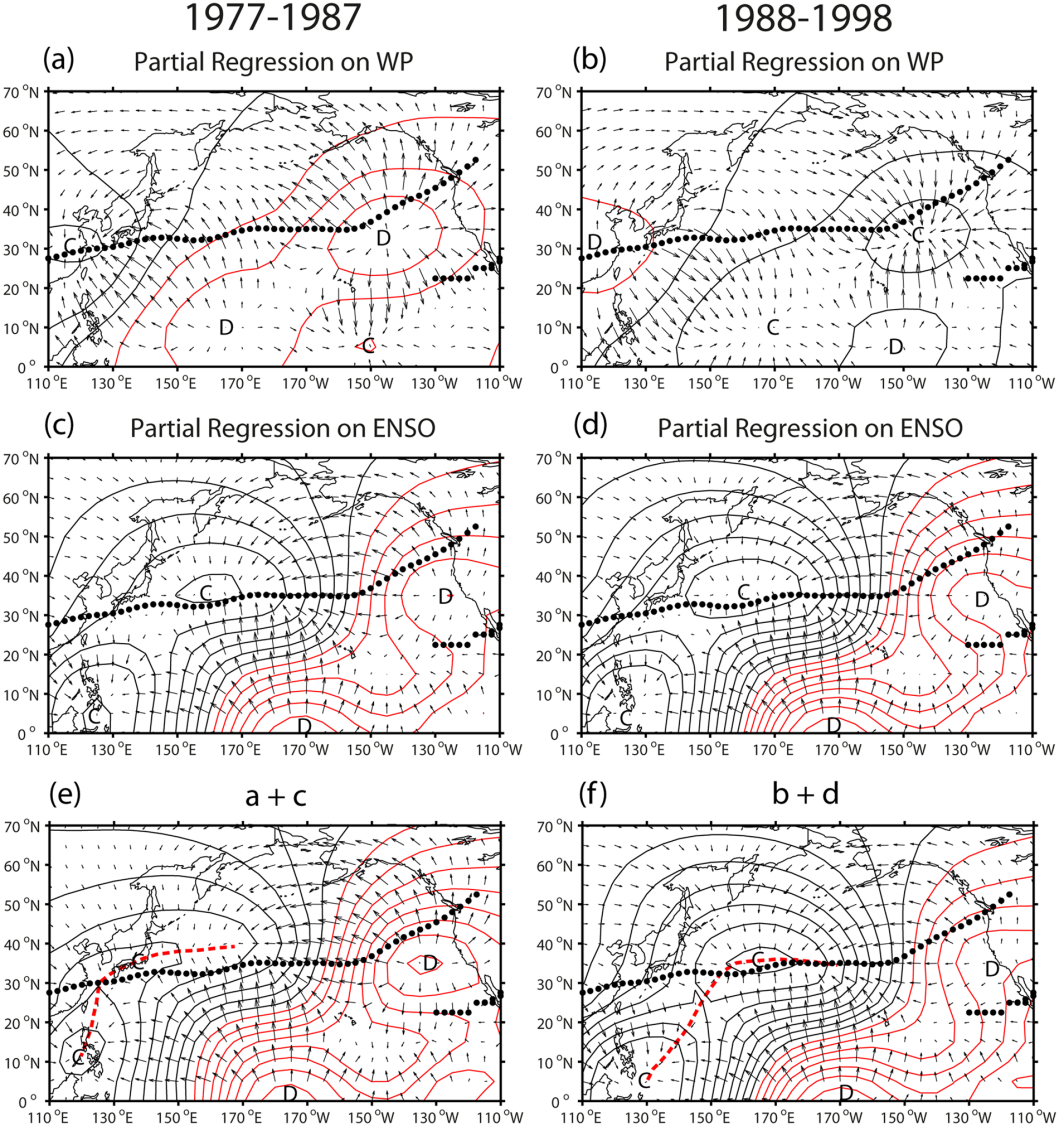
Partial regression of the divergent wind and velocity potential onto the WP and ENSO indices. (**a**,**c**) WP and ENSO regressions for 1977–1987. (**b**,**d**) Same as (**a**,**c**) but for 1988–1998. (**e**,**f**) Sum of the WP and ENSO regressions for the two epochs. Centers of convergence and divergence are marked as C and D, respectively, with the climatological jet axis indicated by black dots. Red dashed lines in (**e**,**f**) represent extended regions of convergence. The superimposed maps are created by the MATLAB mapping package M_MAP v1.4 h using the m_coast function (https://www.eoas.ubc.ca/~rich/map.html).

Although the RWS analysis gives a complete description of the upper-level divergence (Fig. 4) and corresponding meridional overturning circulation in the western Pacific (Fig. 5), the exact partition of ENSO and WP impacts on the RWS is not easy to delineate. This is because each EOF mode of RWS is correlated with multiple climate indices (ENSO, WP, PNA), although ENSO (WP) is best correlated with the EOF1 (EOF2) of RWS. If we consider only the predominant stretching term of RWS, which is mostly a function of anomalous upper-level divergence, a simpler and more comprehensible picture can be obtained, but at the expense of being slightly incomplete. Figure 7 presents the partial regression of the divergent wind and velocity potential onto the WP and ENSO indices as well as their sum, separately for the two epochs before and after the 1988 regime shift. The partial regression coefficients representing the specific part of each index are obtained using a multiple regression (https://fr.mathworks.com/help/stats/regress.html?requestedDomain = true), and the epoch-mean regression is obtained by multiplying the results by the epoch-mean standardized WP or ENSO index (see Methods). For the WP regression, there is for 1977–1987 (Fig. 7a) a prominent subtropical divergence in the jet exit region (35°N, 145°W), which is paired with a prominent subtropical convergence in the jet entrance region (30°N, 115°E). There is also a pair of secondary tropical divergence (10°N, 170°E) and convergence (5°N, 150°W). The jet entrance region over the East Asian coast stands out as a pronounced convergence zone from both the tropics and extratropics, similarly to the RWS climatology (Fig. 3a). The situation for 1988–1998 (Fig. 7b) is completely reversed, with a pronounced divergence in the jet entrance region and convergence in the jet exit region. For the ENSO regression (Fig. 7c,d), there is a strong divergence in the central equatorial Pacific at 170°W, which is paired with both a tropical convergence (Walker cell) south of Philippines (5°N, 125°E) and a subtropical convergence (local Hadley cell) in the central western Pacific (35°N, 160°E), which is in turn paired with a secondary subtropical divergence near the west coast of North America (35°N, 120°W). For the ENSO regression, there appears no significant difference between the two epochs, consequence of an insignificant difference of the epoch-mean ENSO index (Fig. 3c). The sum of the WP and ENSO regressions shows a great contrast in the jet entrance region between the two epochs. For 1977–1987 (Fig. 7e), aside from the Walker cell-associated tropical convergence over Philippines (10°N, 120°E), there appears in the western Pacific an eastwardly-elongated convergence (red dashed line), starting from East China Sea (30°N, 125°E), passing over southern Japan, and extending towards the central Pacific. This upper-level convergence concentrated in the jet entrance region, which is largely due to the upper-level convergence associated with the negative phase of the WP (Fig. 7a), acts to enhance the subtropical bridge pillar. For 1988–1998 (Fig. 7f), on the contrary, no convergence is found in the jet entrance region and the local Hadley cell-associated convergence is located to the east of 150°E. This is partly due to the upper-level divergence associated with the positive phase of the WP (Fig. 7b). Consequently, for the post-regime shift epoch, no divergent winds originating from the tropics reach the jet entrance region west of 150°E (see the red dashed line in Fig. 7f), thus acting to destroy the subtropical bridge pillar. Taken together, these results are practically same as those of the RWS analysis, nicely supporting the original concept of the nonstationary interference of the RWS-associated meridional overturning circulation as a key mechanism responsible for the regime-dependent nonstationary ENSO-WP relationship.

## Methods

### Data

Meteorological parameters used in this study are from the National Centers for Environmental Prediction–National Center for Atmospheric Research (NCEP–NCAR) Reanalysis data^27^. All analyses except for STA are based on monthly data, while daily data are used for STA analysis. The results are then averaged for the 3-month winter season (December through February) before any subsequent statistical analyses.

### Barotropic vorticity equation for Rossby wave source (RWS)

Linear barotropic vorticity equation for the conservation of absolute vorticity $\zeta$ can be written as^3^:

1 $$\,\,\partial \zeta /\partial t+\nabla \cdot ({\boldsymbol{V}}\zeta )=0$$

where $\zeta$ = $\xi +f\,$($\xi$ = relative vorticity, *f* = planetary vorticity). Decompose ***V*** into rotational (${{\boldsymbol{V}}}_{\psi }$) and divergent component (${{\boldsymbol{V}}}_{\chi }$), ${\boldsymbol{V}}=\,{{\boldsymbol{V}}}_{\psi }+\,{{\boldsymbol{V}}}_{\chi }$, then (1) becomes, after arrangement:

2 $$\partial \zeta /\partial t+{{\boldsymbol{V}}}_{\psi }\cdot \nabla \zeta =-{{\boldsymbol{V}}}_{\chi }\cdot \nabla \zeta -\zeta \nabla \cdot {{\boldsymbol{V}}}_{\chi }.$$

The RWS is defined as the right hand side of (2), which can be interpreted as a forcing of the divergent wind for redistributing the absolute vorticity by the rotational velocity field, the left hand side of (2). The RWS has two components: the advection of absolute vorticity by divergent flow $(-{{\boldsymbol{V}}}_{\chi }\cdot \nabla \zeta )$ and the generation of vorticity by divergence or stretching $(-\zeta \nabla \cdot {{\boldsymbol{V}}}_{\chi })$. Finally, the anomalous RWS terms we have calculated are:

$$RWS\text{'}=-\,{{\boldsymbol{V}}}_{\chi }^{^{\prime} }\cdot \nabla \bar{\zeta }-\overline{\,{{\boldsymbol{V}}}_{\chi }}\nabla \zeta \text{'}-\bar{{\rm{\zeta }}}\nabla \cdot {{\boldsymbol{V}}}_{\chi }^{^{\prime} }-\,\zeta \text{'}\nabla \cdot \overline{\,{{\boldsymbol{V}}}_{\chi }},$$

where the over-bar and prime stand for the climatological mean (over the total analysis period 1970–2015) and anomaly from the climatology, respectively.

### Epoch-mean regression

The standard linear regression of a dependent variable *y* onto an independent variable *x* can be written as *y* = *ax*, where both variables are assumed to be centered about their respective time-means. The regression coefficient *a* represents the change of *y* per unit standard deviation (STD) change of *x* for the entire analysis period. The epoch-mean regression of *y* for a limited time period is then obtained by multiplying *a* with the epoch-mean standardized *x*. Note that all PC and index time series have previously been standardized by dividing the centered time series by their respective STDs.

### Data availability

The WP and PNA indices are available from the NOAA/NCEP/Climate Prediction Center website (http://www.cpc.ncep.noaa.gov/data/teledoc/telecontents.shtml), while the ENSO index we used is the multivariate ENSO index (MEI) from the NOAA/Earth System Research Laboratory website (http://www.esrl.noaa.gov/psd/enso/mei/).
